# Chondrocyte Differentiation of Human Endometrial Gland-Derived MSCs in Layered Cell Sheets

**DOI:** 10.1155/2013/359109

**Published:** 2013-11-18

**Authors:** Waki Sekine, Yuji Haraguchi, Tatsuya Shimizu, Masayuki Yamato, Akihiro Umezawa, Teruo Okano

**Affiliations:** ^1^Institute of Advanced Biomedical Engineering and Science, TWIns, Tokyo Women's Medical University, 8-1 Kawada-cho, Shinjuku-ku, Tokyo 162-8666, Japan; ^2^Department of Reproductive Biology, National Center for Child Health and Development, 2-10-1 Okura, Setagaya-ku, Tokyo 157-8535, Japan

## Abstract

Recently, regenerative medicine using engineered three-dimensional (3D) tissues has been focused. In the fields of cell therapy and regenerative medicine, mesenchymal stem cells (MSCs) are attractive autologous cell sources. While, in bioengineered tissues, a 3D environment may affect the differentiation of the stem cells, little is known regarding the effect of 3D environment on cellular differentiation. In this study, MSC differentiation in *in vitro* 3D tissue models was assessed by human endometrial gland-derived MSCs (hEMSCs) and cell sheet technology. hEMSC sheets were layered into cell-dense 3D tissues and were cultured on porous membranes. The tissue sections revealed that chondrocyte-like cells were found within the multilayered cell sheets even at 24 h after layering. Immunostainings of chondrospecific markers were positive within those cell sheet constructs. In addition, sulfated glycosaminoglycan accumulation within the tissues increased in proportion to the numbers of layered cell sheets. The findings suggested that a high cell density and hypoxic environment in 3D tissues by layering cell sheets might accelerate a rapid differentiation of hEMSCs into chondrocytes without the help of chondro-differentiation reagents. These tissue models using cell sheets would give new insights to stem cell differentiation in 3D environment and contribute to the future application of stem cells to cartilage regenerative therapy.

## 1. Introduction

Cell-based regenerative therapy has a potential for treating diseased/defective tissues and organs that are unable to be cured by conventional medical treatments including medicines and surgeries. Regenerative medicine using artificial tissue fabricated by scaffold-based tissue engineering has appeared as the second-generation therapy, and the clinical trials have been performed [1–4]. Scaffold-based tissue engineering has been currently based on a concept that three-dimensional (3D) biomaterials including polycaprolactone, collagen, gelatin, and so forth are used as an alternative to extracellular matrix (ECM) and cells are seeded into the materials.

Our laboratory has developed a scaffolds-free tissue engineering, “cell sheet engineering,” using temperature-responsive culture surfaces, which can control the attachment and detachment of living cells by simple temperature changes [5–7]. Three-dimensional cell-sheet constructs fabricated by the technology are clearly cell-dense, and their functional junctions are tightly formed among cells within the tissues [8–10]. For example, cardiomyocytes in engineered 3D myocardial tissues coupled electrically and functionally via gap junctions, and the tissues can beat synchronously like the real heart [8, 10]. In addition, the authors have recently found that thicker tissue (the thickness of sextuple-layered cell sheets: more than 100 *μ*m) was able to be fabricated by a 3D-tissue culture method on porous membrane, where the thickness limitation of viable tissue depends on oxygen and nutrient gradients could be avoided [9]. The circumstances of the thicker cell-dense 3D tissue culture are thought to be quite different from those of monolayer two-dimensional (2D) culture.

 Mesenchymal stem cells (MSCs), which are a type of somatic stem cells, are an attractive autologous cell source for cell-based regenerative therapy, since they have a strong ability to proliferate actively *in vitro* and differentiate into various cells including chondrocytes, osteocytes, adipocytes, skeletal myoblasts, cardiomyocytes, and so forth, with the treatments of optimal bioactive factors including cytokines or their surrounded circumstances [11–17]. In addition, those cells can be isolated easily from various tissues including bone marrow, adipose tissue, umbilical cord, amniotic fluid, peripheral blood, and so forth. Recently, some stem cells, which express surface antigens similar to those of bone marrow-derived MSCs, are isolated from human menstrual blood and endometrial gland [18].

 Cells within a 3D culture system are reported to be significantly different from those in a 2D culture system in terms of their morphology, cell-cell interactions, surrounding ECM, proliferation rates, differentiation, and so forth. [19–21]. These differences may be affected by their different circumstances of oxygen, nutrients, growth factors, cell-cell and cell-matrix interactions, and so forth. Although, in 2D culture, oxygen tension, and the concentrations of nutrients and growth factors are unusually high, in 3D culture, cells are subject to multiple stimuli, namely, cytokines, growth factors, and proteins secreted from surrounding cells [22]. Cells are also affected by biochemical and mechanical interactions with ECM as well as direct cell-cell contacts [22].

 In this study, cell-dense thicker 3D tissues were fabricated from human endometrial gland-derived MSC (hEMSC) sheets, and the differentiation of the stem cells within the tissue was assessed and analyzed.

## 2. Materials and Methods

### 2.1. Preparation and Layering of hEMSC Sheets

hEMSCs, which showed an adherent spindle-shape morphology, were cultured in Dulbecco's modified Eagle's medium (DMEM) (Sigma-Aldrich, St. Louis, MO, USA) supplemented with 10% fetal bovine serum (FBS) (Japan Bio Serum, Nagoya), 1% penicillin/streptomycin (Invitrogen, Carlsbad, CA, USA) [9, 18, 23]. hEMSC sheets were fabricated as previous reports [9, 10, 23]. Briefly, hEMSCs (1.0 × 10^6^ cells) were cultured on a 35 mm diameter temperature-responsive culture dish (Upcell, CellSeed, Tokyo, Japan) for 4 days at 37°C, and the culture dish was placed in a CO_2_ incubator at 20°C. A hEMSC sheet detached itself spontaneously within 30 min. The cell sheets were layered on a cell-culture insert (Becton, Dickinson and Company, Franklin Lakes, NJ, USA) having a track-etched PET membrane (the membrane pore size: 1 *μ*m), because a previous report has shown that the cultivation of layered cell sheets on the porous membranes induces the improvement of cell viability and the fabrication of thicker tissues than that on normal culture surfaces [9]. The insert was set in a 6-well cell-culture insert companion plate (Becton, Dickinson and Company). Harvested cell sheets from temperature-responsive culture surfaces were layered on the membrane as described in previous reports [9]. Briefly, a cell sheet with media was gently aspirated into the tip of a pipette and was transferred onto the membrane, which was pretreated with FBS for more than 30 min. After the first cell sheet was spread, the media were aspirated, and the plate was incubated at 37°C for allowing the cell sheet to fully adhere to the membrane. To layer hEMSC sheets, the second sheet recovered from another temperature-responsive culture dish was layered onto the first cell sheet. By the same fashion, recovered cell sheets were successfully layered up to sextuple layer. The multilayered cell sheets were cultured for 24 h in a humidified atmosphere of 5% CO_2_ at 37°C with 2 mL medium (DMEM supplemented with 10% FBS, 1% penicillin/streptomycin) in both insert and well. 

### 2.2. Histological Analysis

After being incubated for 24 h, multilayered cell sheets on the porous membranes were fixed with 4% paraformaldehyde solution (Muto Pure Chemicals, Tokyo, Japan) at least for 1 day. Specimens were embedded in paraffin, sectioned, and stained with hematoxylin and eosin. Prepared specimens were observed by an optical microscope (ECLIPSE TE2000-U) (Nikon, Tokyo, Japan).

### 2.3. Immunohistochemistry

For detecting chondrocyte differentiation, the deparaffinized sections were also stained with the antibodies of chondrospecific markers: (1) monoclonal anti-human type II collagen (Cosmo Bio, Tokyo, Japan), which were diluted at 1 : 100 and (2) hyaluronan-binding protein (Seikagaku Corporation, Tokyo, Japan), which were diluted at 1 : 200. The detections of the immuneoreactions were enhanced by a CSA ll Biotin-free Tyramide Signal Amplication System (Dako Cytomation, Glostrup, Denmark) according to the manufacturer-suggested protocol. Hematoxylin was used for nuclear counterstaining. Prepared specimens were observed by the optical microscope.

### 2.4. Measurement of Sulfated Glycosaminoglycan Accumulation within Layered hEMSC Sheets

The accumulation of sulfated glycosaminoglycan (sGAG), which is the constituent of ECM in cartilage tissues, within layered hEMSC sheets was quantitated by a commercially available kit (Seikagaku Biobusiness, Tokyo, Japan), which is a colorimetric determination method using 1,9-dimethylmethylene blue.

### 2.5. Data Analysis

Data were expressed as the mean ± SD. Dunnett's test was performed to compare two groups.

## 3. Results and Discussion

### 3.1. Morphological Analysis of the Layered hEMSC Sheets

**Figure 1:**
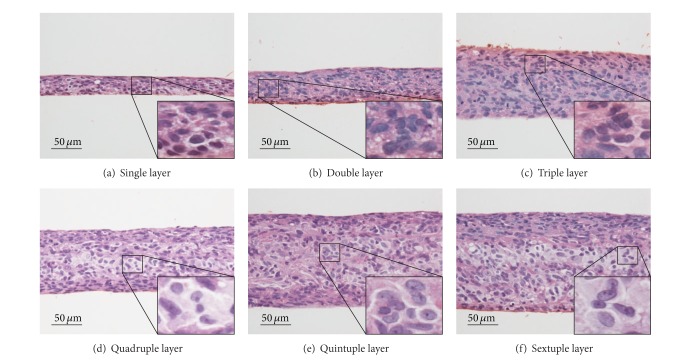
Histological observation of layered human endometrial gland-derived mesenchymal stem cells (hEMSC) sheets after a 24 h cultivation on porous membranes. All microphotographs are the hematoxylin and eosin stained cross-sections of the cell sheets ((a) single-layered cell sheet; (b) double-layered cell sheet; (c) triple-layered cell sheet; (d) quadruple-layered cell sheet; (e) quintuple-layered cell sheet; (f) sextuple-layered cell sheet). Enlarged photographs showed chondrocyte-like cells. Independent three experiments were performed and those experiments showed similar results. The representative photographs were shown in the figure.

**Figure 2:**
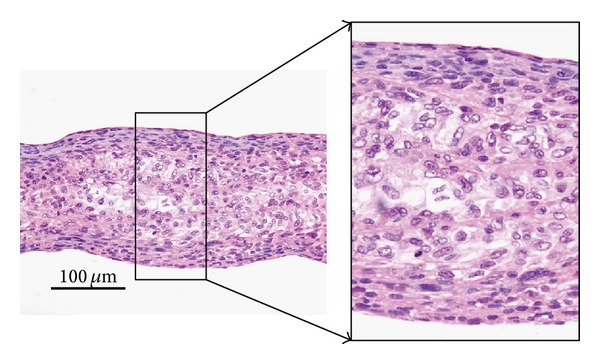
Histological observation of sextuple-layered hEMSC sheets. The left photograph is the cross-sectional observation of sextuple-layered cell sheets at 24 h after layering. The right is an enlarged photograph of the center part of the left photograph.

hEMSC sheets were layered into 3D tissues onto a porous membrane, and the differentiation kinetics of the stem cells within the cell sheet constructs were histologically examined. At 24 h after the cultivation of layered hEMSC sheets, the constructs were observed cross-sectionally. Because, after detaching from temperature-responsive culture dishes, a cell sheet shrunk horizontally due to the cytoskeletal tensile reorganization, a single-layer cell sheet consisted of several cell-layers, and the thickness of the cell sheet became more than 25 *μ*m (Figure 1). With increasing the number of stratified cell sheets, the thicknesses of the layered cell sheets increased as shown in Figure 1. The thicknesses of more than triple-layered cell sheets were more than 100 *μ*m, and those cell layer tissues were clearly cell dense. Some characteristic cells were found within the multilayered cell-sheet constructs and resembled differentiated chondrocytes (Figure 1). Many chondrocyte-like cells were found within more than quadruple-layered cell-sheet constructs (Figures 1 and 2). After the cell sheet layering, hEMSCs might differentiate into chondrocytes very rapidly (24-h cultivation) within the multilayered cell sheet constructs. On the other hand, in single-, double-, triple-layered cell-sheet constructs, similar cell structures were observed (Figure 1).

### 3.2. Expression of Chondrospecific Markers in Layered hEMSC Sheets

**Figure 3:**
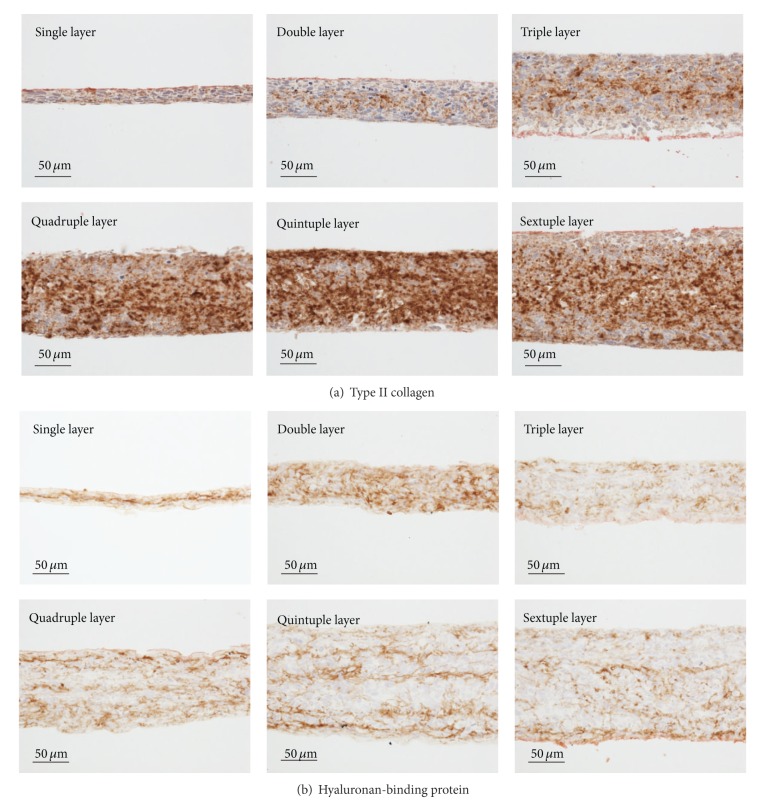
Detection of chondrospecific markers in layered cell sheet constructs at 24 h after layering by immunohistochemistry. Cells in single- and multilayered cell sheet constructs expressed type II collagen (a) and hyaluronan-binding protein (b).

The expressions of chondrospecific markers in cells within 3D cell-sheet tissues fabricated by layering hEMSC sheets were examined by an immunohistological analysis. While, especially, the expression of type II collagen was strongly detected in multilayered (more than quadruple-layered) cell-sheet constructs, in all cell-sheet constructs including a single-layer cell sheet, the expression was detected (Figure 3(a)). Hyaluronan-binding protein was also expressed in all layered cell-sheets constructs (Figure 3(b)). These results confirmed the chondro-differentiation of hEMSCs within the multilayered cell sheet constructs. At the same time, the chondrocyte differentiation was suggested to occur in all cell-sheet constructs including a single-layer cell sheet. While those chondrospecific markers-positive cells were detected in the tissue constructs even at 24 h after the layering without the adding of chondro-differentiation reagents, no further increases were detected by further cultivation (data not shown). Those results were well consistent with that of morphological analysis (data not shown).

**Figure 4:**
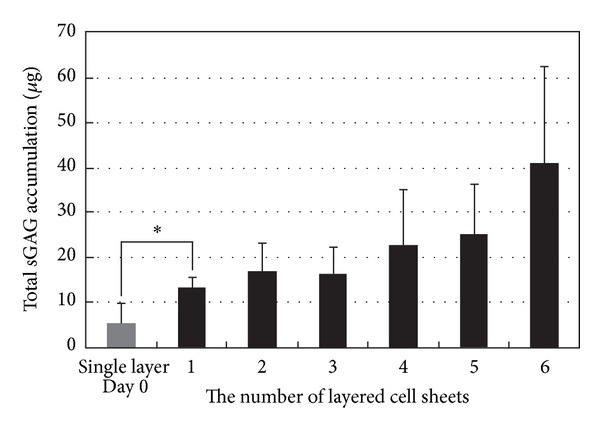
Detection of sulfated glycosaminoglycan (sGAG) accumulation within layered cell sheets at 24 h after the start of cultivation. With increasing the number of stratified cell sheets (from single to sextuple), sGAG accumulation increased. Data are shown as the mean ± SD (*n* = 3). The sGAG accumulations within a cell sheet enhanced during 24-h cultivation after detachment (*P < 0.05).

 sGAG accumulation within the constructs was measured quantitatively. The sGAG accumulations within cell sheets enhanced during a 24 h cultivation after detachment, and with increasing the number of stratified cell sheets (from single to sextuple), total sGAG accumulation tended to increase (Figure 4).

 In this study, *in vitro* chondrocyte differentiation from hEMSCs within 3D tissue constructs fabricated by layering the stem cell sheets was shown. Articular cartilage has a poor ability for self-regeneration after the defective/injury, because it shows a low cell density and has limited blood supply. Various therapies including chondrocyte transplantation and tissue engineering methodology using 3D scaffolds have been performed clinically for regenerating articular cartilage damage [3, 24, 25]. For those therapies, autologous chondrocytes are isolated from the tissues and used as a cell source. At the same time, bone marrow-derived and adipose tissue-derived MSCs were also used as other autologous cell sources. MSCs differentiate into chondrocytes in the transplanted area *in vivo* and the implantation induces good therapeutic effects in animal models, and the therapies using bone marrow-derived MSCs have been clinically performed [17, 26–31]. This study suggested that hEMSCs as well as bone marrow-derived and adipose tissue-derived MSCs should be useful as a cell source for cartilage regenerative medicine. In addition, the chondrocyte differentiation of hEMSCs in 3D tissues fabricated by layering cell sheets was rapid (24 h cultivation after layering). Normally, *in vitro* differentiation from MSCs into chondrocytes is taken about for approximately three weeks by a conventional method [32]. Transplantation after the short term *in vitro* cultivation of layered hEMSC sheets into defective cartilage tissues might induce a stronger therapeutic effect than that of undifferentiated stem cells. On the other hand, although the expressions of chondrospecific markers were detected in the layered cell-sheet constructs even at 24 h, no further increases were detected by further cultivation (data not shown).

 Cell sheet technology has now been applied to the regeneration of various damaged tissues, and clinical trials have been ready performed in several tissues including cornea epithelial, myocardial, esophageal, and periodontal tissues [33–37]. Because confluent cells on a temperature-responsive culture surface can be harvested as an intact and contiguous cell sheet by simply reducing the temperature without protease treatment, cell-cell junctions and ECM components mediating cell adhesion, which are susceptible to protease treatment, are preserved intact in the cell sheet [38–40]. Because cell sheets maintain cell-cell junctions and ECM components, cell-dense and functional 3D tissues can be easily fabricated by simply layering cell sheets without any scaffold, and the engineered tissues can also adhere directly to the host tissues without suturing [41]. More recently, the technology has been successfully applied to repair/regenerate the damaged cartilage tissue [42–46] and the clinical study has also been started. In *in vivo* and *ex vivo* experiments, multilayered human articular chondrocyte sheets were found to adhere firmly to rabbit and porcine cartilage tissues [42, 45]. The higher expressions of cell adhesion molecules, fibronectin and integrin *α*10 were detected in the multilayered chondrocyte sheets than those in monolayer chondrocytes [42, 44]. Layered chondrocyte sheets, which express highly those cell adhesion molecules, on temperature-responsive culture surfaces can be harvested by simply reducing culture temperature. In addition, scanning electron microscopical observation showed that the basal surface of the chondrocyte sheet had a completely different surface texture from that of the top of the sheet, and in other words, compared with the top surface, the bottom surface showed a smooth arrangement of the accumulated ECM with a parallel pattern [45]. The good adhesiveness of layered chondrocyte sheets onto the tissues might be related to the preservation of those molecules as well as the smooth arrangement of the basal surface, which more resembles normal cartilage surface than the top side of the cell sheet. Furthermore, while human articular chondrocytes is known to lose their chondrocyte phenotype during 2D cultivation [47], in the multilayered chondrocyte sheets, the expressions of chondrospecific markers, namely, type II collagen, SOX9, Aggrecan, are significantly increased in comparison to those in 2D cultured chondrocytes or in a single chondrocyte sheet, indicating that a multilayered chondrocyte sheet has a natural cartilage phenotype [42, 44]. The maintenance of chondrogenic phenotype might be related to the 3D environment of layered cell sheets. This study showed that an environment within 3D tissues fabricated by layering cell sheets might accelerate the differentiation of hEMSCs into chondrocytes. In the original and general chondrocyte differentiation methods from MSCs, the cells are collected into high cell-density pellets to induce a round shape and treated with cytokines/reagents described above [11–13]. In this study, the high cell density and hypoxic environment within 3D tissues might promote the chondrocyte differentiation of MSCs and the maintenance of chondrogenic phenotypes. *In vitro* cultivation under hypoxia has been reported to promote the restoration of chondrogenic phenotypes in human articular chondrocytes and enhance the chondrogenic differentiation of human bone marrow-derived MSCs [48, 49]. A previous report using a fiber-optical oxygen microsensor has showed that the oxygen concentrations between single- or multilayered EMSC sheets, and the insert membrane are quickly decreased, suggesting a hypoxia condition within layered cell sheets including single-layer cell sheets, whose thicknesses are more than 25 *μ*m [9]. The elucidation of the detail molecular mechanisms of chondrocyte differentiation within 3D tissues should contribute to the establishment of novel and effective chondrocyte differentiation methods from MSCs and the fabrication of valuable 3D cartilage tissue. Layered cell sheets containing chondrocytes differentiated from hEMSCs on temperature-responsive culture surfaces were able to be harvested with preserving cell adhesive molecules on the cell surfaces and would contribute to cartilage tissue engineering and regenerative medicine.

## 4. Conclusion

This study assessed and analyzed human EMSC differentiation *in vitro* 3D tissue models using cell sheet technology. This study suggested that a high cell density and hypoxic environment in 3D tissues fabricated by layering cell sheets might accelerate a rapid differentiation of MSCs into chondrocytes without the help of chondro-differentiation reagents. These 3D tissue models using cell sheets would give new insights to stem cell differentiation in 3D environment and contribute to the future application of the stem cells to cartilage regenerative therapy.
